# Effects of Seasonal Births and Predation on Disease Spread

**DOI:** 10.1007/s11538-026-01697-1

**Published:** 2026-07-02

**Authors:** Allison J. Introne, Leah B. Shaw

**Affiliations:** 1 Department of Mathematics, William & Mary, Williamsburg, 23187 VA USA; 2 Biomathematics Graduate Program, North Carolina State University, Raleigh, 27607 NC USA; 3 Department of Mathematics, North Carolina State University, Raleigh, 27607 NC USA

**Keywords:** Seasonal infection, Density dependent predation, Pulsed births, Flow-kick dynamics

## Abstract

For many organisms, births occur in seasonal pulses. When predation on an organism is density-dependent, seasonal population changes also change the per capita predation rate. This in turn can affect disease dynamics in the system. We use a Susceptible-Infected (SI) model to study the effects of seasonal births and nonlinear predation on the spread of infection in a population over time. Motivated by open marine systems, we assume that the total birth or recruitment rate is density-independent and that the predator population is constant. We compare models with constant year-round births and seasonally pulsed births as well as linear and hyperbolic predation functions. We calculate the basic reproductive number and use numerical simulations to examine system behavior when infection is endemic. When predation depends linearly on prey populations, seasonality of births does not affect the average infection level or disease transmissibility. When predation rates saturate, as in systems with hyperbolic predation, higher disease transmissibility and endemic infection levels result. This effect can be exacerbated by seasonality of births, especially at moderate population levels.

## Introduction

Some infectious diseases exhibit seasonal changes in prevalence, which are driven by a variety of mechanisms. Seasonal changes in rainfall or temperature affect factors such as vector abundance and pathogen growth and transmission, which can lead to seasonal outbreaks of diseases like malaria (Hoshen and Morse 2004), dengue (Watts et al. 1987), and cholera (Lipp et al. 2002). Seasonal changes in host behavior, such as social aggregation, contribute to outbreaks of measles in humans (Finkenstädt and Grenfell 2000) and bacterial conjunctivitis in house finches (Hosseini et al. 2004). Seasonal wildlife births lead to periodic outbreaks by suddenly increasing susceptible host abundance (Altizer et al. 2006). While natural systems are typically influenced by multiple seasonal drivers, we will focus on the effects of seasonal births on disease dynamics.

Many organisms reproduce in seasonal birth pulses in which most of the new individuals are born within a short amount of time periodically (Peel et al. 2014). Multiple diseases have been studied that are believed to be affected by seasonal host births (Altizer et al. 2006). A motivating example is *Hematodinium* sp. infections in blue crabs. Blue crabs exhibit seasonality in the timing of births and recruitment of new individuals (van Montfrans et al. 1990). Messick and Shields (2000) researched the environmental and host factors that affect *Hematodinium* sp. infection prevalence and found that infection prevalence in coastal bays of Maryland peaked in autumn not long after the start of recruitment and declined rapidly in the winter, leading to periodic outbreaks of infection. Since juvenile crabs are more susceptible to the infections compared to mature crabs and an outbreak of *Hematodinium* sp. infection occurred shortly after the influx of new juveniles each year, it was hypothesized that annual birth pulses contribute to the seasonality of the disease (Messick and Shields 2000). We are particularly interested in diseases where individuals do not typically recover. *Hematodinium* sp. infections in blue crabs are highly pathogenic and associated with high mortality rates, limiting the potential for recovery (Messick and Shields 2000; Shields and Squyars 2000).

The blue crab-*Hematodinium* system, and marine systems more broadly, inspired several modeling choices. We assume that the birth rate is independent of population, corresponding to recruitment of marine larvae that were spawned at another location. Source-sink dynamics are common in marine populations (Tamaki 2023). Thus, what we term a “birth” rate may be considered a rate of immigration to a sink population. Using a population-independent recruitment rate allows us to focus on the role of birth timing without considering how the resulting population feeds back to alter subsequent birth rates. We also assume a generalist predator whose population is not affected by the prey population. Generalist predators are particularly important in marine systems (Duffy and Hay 2001; Dolecal and Long 2014). When prey populations vary seasonally, as with pelagic coelenterates, the diet of generalist predators contains a lower percentage of that prey when it is less abundant (Arai 2005). Complicating matters, blue crabs are frequently cannibalized by conspecifics (Hines 2007; Lipcius et al. 2007), meaning that death by predation may not directly lead to increases of the predatory population. For simplicity, we instead treat the predator population as a parameter. Natural systems are influenced by a multitude of factors, but we chose to model seasonal births independent of other components, with the exception of predation by a constant predator population, to identify their contribution to outbreaks of disease, particularly in a species with high mortality losses to predators.

The model in this paper is a Susceptible-Infected (SI) compartmental model. Several studies include periodic components in their compartmental models corresponding to different processes, such as pulsed treatment coverage (Walter and Lion 2021) and seasonal fishing (Tang and Chen 2004). We are interested in modeling seasonal birth pulses and do so through the use of impulsive differential equations in which susceptible individuals are added in a pulse each year. Previous studies have examined models with seasonal births, including density-independent (Cao and Jin 2007), constant per capita (Zhang et al. 2009), logistic (Zhang et al. 2008), and general negative density-dependent (Roberts and Kao 1998) birth rates. Peel et al. (2014) modeled three different periodic birth functions to look at the impact on infection persistence and determined that a periodic Gaussian function produced the most realistic representation of seasonal births compared to a cosine or step function. The researchers also found that the synchrony of the birth pulse played a role in pathogen persistence. Birth pulses in which individuals were born within a shorter period of time led to larger oscillations in populations, making it more likely for a pathogen to go extinct (Peel et al. 2014). Hosseini et al. (2004) constructed a model of *Mycoplasma gallisepticum* infections in house finches to determine the role of seasonal births and seasonal social aggregation in infection spread. They used a birth rate function tailored to data on avian breeding patterns. They found that seasonal drivers affected the disease dynamics and that the combination of seasonal functions led to semi-annual peaks in infection prevalence. Various models have confirmed that seasonal host births, represented in a variety of ways, do affect disease dynamics. This motivates our interest in studying pulsed births as opposed to constant births. Since we are modeling instantaneous birth pulses, we use a Dirac delta function to model seasonal births. This allows more analysis than with the realistic periodic Gaussian function studied in (Peel et al. 2014).

In addition to seasonal births, predation is another factor that influences disease dynamics. Studies have found that, in some cases, removal of a predator can lead to decreased prey populations in the presence of disease. This may be due to the lack of removal of infected individuals by predators, which causes the prey population to die from the disease (Hethcote et al. 2004). Predation can be modeled with functional responses, which are functions of the prey density that describe the rate at which prey are consumed (Holling 1959, 1965). The predation rate is often chosen to depend linearly on the prey population for simplicity. Another common and more realistic predation functional response is saturating predation. In this case, predators become satiated as the prey population increases (Hall et al. 2005). Hall et al. (2005) analyzed an SI model with saturating predation to determine how predation impacts infection persistence. They found that saturating predation along with preferential predation affected whether the predator caused extinction of the pathogen or the combination of infection and predation led the host species to go extinct. Population changes throughout the year can lead to changes in the per capita predation rates which, in turn, can affect disease dynamics. Similar to (Hall et al. 2005), we consider a saturating per capita predation rate (Holling type II, Holling (1959)), but we build upon previous work by studying the combined effect of different predation functional responses and seasonal birth pulses.

Since we treat the predator population as a parameter, our predation term could instead be interpreted as natural mortality with negative density-dependence. Natural mortality is more commonly assumed to have positive density-dependence, as occurs with competition. In a model with pulsed or continuous births, Roberts and Kao (1998) assumed that per capita mortality was an increasing function of population. However, negative density-dependent mortality can occur for various reasons; for example, dense aggregations of mussels have reduced mortality from cold winters and physical disturbances as well as predation (Bertness and Grosholz 1985; Van De Koppel et al. 2008; Liu et al. 2012).

The SI model discussed in this paper is unique in combining a seasonal birth function and different predation functional responses. In Section 2 we define four model versions, including constant and pulsed births, and linear and hyperbolic predation. We calculate the basic reproductive number for each model version in Section 3 and discuss the endemic behavior in Section 4.

## Model

We model a population with *S* susceptible individuals, *I* infected individuals, and total population $N=S+I$. We first consider a constant per capita natural mortality rate *m*:

1 $$\begin{aligned} \frac{\textrm{d}S}{\textrm{d}t}&=\mathcal {b}(t)-\beta IS-mS \end{aligned}$$

2 $$\begin{aligned} \frac{\textrm{d}I}{\textrm{d}t}&=\beta IS-\mu I-mI, \end{aligned}$$

where *t* is the time in years, $\mathcal {b}(t)$ is the birth rate, $\beta$ is the transmission rate, and $\mu$ is the per capita disease-induced mortality rate.

We compare two options for the birth rate $\mathcal {b}(t)$. First, births can occur at a constant rate,

$$\begin{aligned} \mathcal {b}(t)=b. \end{aligned}$$

Second, births can occur in annual pulses,

$$\begin{aligned} \mathcal {b}(t)=b\sum _{n=-\infty }^{\infty }\delta (t-n), \end{aligned}$$

where $\delta (t)$ is the Dirac delta function. This assumes that new susceptible individuals are added instantaneously in pulses at integer values of time at an average rate of *b* births per year. The pulsed option can equivalently be expressed as

$$\begin{aligned} \frac{\textrm{d}S}{\textrm{d}t}&=-\beta IS-mS, \\ \frac{\textrm{d}I}{\textrm{d}t}&=\beta IS-\mu I-mI, \,\, t\ne n \\ S(n^+)&=S(n)+b, \\ I(n^+)&=I(n), \end{aligned}$$

where $n\in \mathbb {Z}$. With either birth option, we assume that the average birth rate *b* is independent of the population size. An example of this in nature is recruitment of larvae into marine populations. The number of larvae that arrive in the system after dispersal often does not depend strongly on the number of organisms in the local population that the larvae join (Szuwalski et al. 2015).

**Fig. 1:**
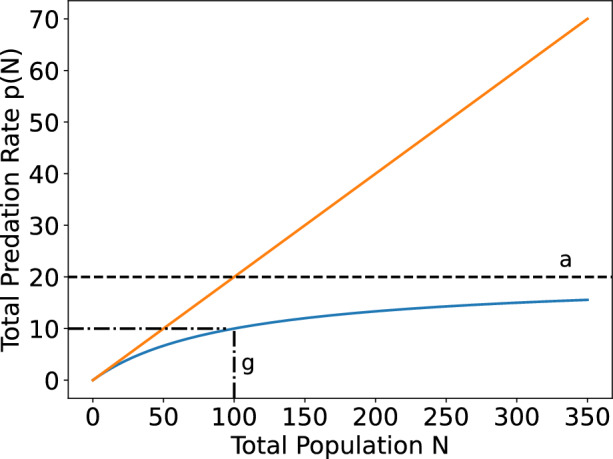
Linear and hyperbolic functional responses versus total population. Predation rate $p(N)=mN$ is linear predation (orange) while $p(N)=\frac{aN}{g+N}$ is hyperbolic predation (blue). Parameters: $m = 0.2$, $a = 20$, $g = 100$. (color figure online)

We assume that most natural mortality is due to predation. Constant per capita natural mortality leads to linear predation terms in Eqs. (1)-(2). To study the effect of predator functional response, we also consider hyperbolic predation. Here, the predators are limited in the number of individuals they can consume, causing the predation rate to saturate at high prey density (Holling 1959). We use total predation rate

$$\begin{aligned} \frac{a(S+cI)}{g+(S+cI)}, \end{aligned}$$

where *a* is the maximum predation rate, *g* is the half-saturation constant or the prey population at half the maximum predation rate, and *c* determines the amount of predator preference for infected individuals. Fig. 1 compares the two predator functional responses.

Selective predation on both diseased and healthy individuals has been observed. For example, studies have found evidence of increased predation on mule deer infected with chronic wasting disease (Krumm et al. 2010), red grouses parasitized by *Trichostrongylus tenuis* (Hudson et al. 1992), *Daphnia dentifera* infected with *Metschnikowia bicuspidata* or *Spirobascillus cienkowskii* (Duffy and Hall 2008), and killifish parasitized by *Euhaplorchis californiensis* (Lafferty and Morris 1996). Butler et al. (2014) found higher predation and cannibalism on blue crabs infected with *Hematodinium perezi*. However, more recent work has found that in more complex nursery habitats, infected juvenile crabs are cannibalized less than healthy crabs, likely due to behavioral changes caused by infection (Guennouni 2025). In our model, if $c=1$, we assume each susceptible or infected individual is equally likely to die from predation. If $c>1$, we assume there is more predation overall and infected individuals are more likely to be preyed upon. Predators are not modeled explicitly, so we are assuming a fixed number of predators independent of time or prey population. With hyperbolic predation, the system of equations is

3 $$\begin{aligned} \frac{\textrm{d}S}{\textrm{d}t}&=\mathcal {b}(t)-\beta IS-\frac{aS}{g+S+cI} \end{aligned}$$

4 $$\begin{aligned} \frac{\textrm{d}I}{\textrm{d}t}&=\beta IS-\mu I-\frac{acI}{g+S+cI}, \end{aligned}$$

where we split the predation mortality proportionally between susceptible and infected individuals, weighting predation on infected individuals by *c*.

We include several restrictions on parameters. We use $a/g=m$ so the hyperbolic predation rate approaches the linear predation rate at small populations (Fig. 1). Additionally, the maximum predation rate *a* is set to be larger than the birth rate: $a>b$. This maintains a finite population via predation even in the absence of disease mortality. Most other parameters are used as bifurcation parameters. Note that we could rescale the population variables to dimensionless units of *b*/*m*, so *b* and *m* need not be independently varied.

**Fig. 2:**
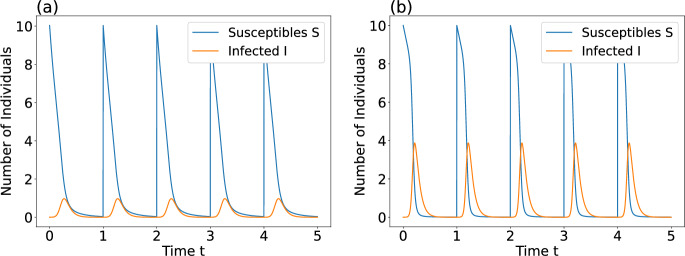
Numerical solution of the linear predation model (**a**) and hyperbolic predation model (**b**) with pulsed births. Parameters: $\beta$ = 8, $\mu$ = 10, $m = 4$, $a = 20$, $g = 5$, $b = 10$, $c=1$

We numerically integrate the differential equations in Python using SciPy’s solve_ivp function. For the pulsed birth case, each year is integrated separately. For example, we implement Eq. (1) by integrating

$$\begin{aligned} \frac{\textrm{d}S}{\textrm{d}t}=-\beta IS-mS \end{aligned}$$

throughout the year. At the end of each year, *b* births are added to the susceptible population, and this new susceptible value along with the final number of infected individuals are used as initial conditions for the next year. Fig. 2 shows sample period 1 solutions resulting from pulsed births. The susceptible population jumps by *b* at each integer time when new births are added instantaneously. Longer period behavior can also occur; see Sect. 4.

## Basic Reproductive Number

To determine when the disease-free state is unstable, we calculate the basic reproductive number $R_0$ using next generation matrix methods (van den Driessche and Watmough 2002; Wang and Zhao 2008) for all four combinations of the following: constant versus pulsed births, and linear versus hyperbolic predation.

The models have two compartments: susceptible and infected. Let $\boldsymbol{x}=(I,S)$ be the number of individuals in the infected and susceptible compartment, respectively. We use two different vector functions to represent the transfer of individuals. $\mathscr {F}(\boldsymbol{x})$ is the rate of new infections appearing in each compartment and $\mathscr {V}(\boldsymbol{x})$ is all other transfers of individuals in and out of the compartments. Given these two functions, our model can be written as $\frac{\textrm{d}\boldsymbol{x}}{\textrm{d}t}=\mathscr {F}(\boldsymbol{x})-\mathscr {V}(\boldsymbol{x})$.

### Linear Predation

We use the next generation matrix technique established by van den Driessche and Watmough (2002) to calculate $R_0$ for the constant birth model with linear predation, which satisfies the assumptions outlined by van den Driessche and Watmough (2002). Evaluating at the disease-free equilibrium (DFE) solution $\boldsymbol{x}_0=(0,S_0)=(0,b/m)$, we find that

$$\begin{aligned} \boldsymbol{F}= \begin{bmatrix}\frac{\partial \mathscr {F}_1}{\partial I}(\boldsymbol{x}_0)\end{bmatrix} = \begin{bmatrix}\beta S_0\end{bmatrix} = \begin{bmatrix}\beta b/m \end{bmatrix} \text { and } \boldsymbol{V}=\begin{bmatrix}\frac{\partial \mathscr {V}_1}{\partial I}(\boldsymbol{x}_0)\end{bmatrix}=\begin{bmatrix}\mu +m\end{bmatrix}. \end{aligned}$$

Because $\boldsymbol{F}$ and $\boldsymbol{V}$ are $(1\times 1)$ matrices, $R_0=\boldsymbol{FV}^{-1}$. So, for the constant birth linear predation model, we find that

5 $$\begin{aligned} R_0=\frac{\beta b}{m(\mu +m)}. \end{aligned}$$

Increasing the birth or transmission rate increases $R_0$, while increasing mortality decreases $R_0$, as one would expect.

We calculate $R_0$ for the pulsed birth model using the technique established by Wang and Zhao (2008). Browne et al. (2015) have argued that this approach applies to piecewise continuous systems, making it suitable for our model. Clearly, the model will approach the disease-free subspace in the absence of new infections. We also show below that there is a period 1 disease-free solution that is stable in the disease-free subspace. Thus, our model satisfies the necessary assumptions of Wang and Zhao (2008).

To find the disease-free periodic solution to Eq. (1), $S_0(t)$, notice that the susceptible population experiences exponential decay, $S_0(t)=Ce^{-mt}$, for $0\le t<1$. When $t=1$, the birth pulse occurs. For a period 1 solution, we have the periodicity constraint $S_0(1)=Ce^{-m}+b=S_0(0)$. Solving for *C*, we obtain

6 $$\begin{aligned} S_0(t) = \frac{be^{-mt}}{1-e^{-m}} \,\,\,\,\,\,\, 0\le t<1, \end{aligned}$$

which then repeats with a 1 year period. Similarly, an arbitrary susceptible population $S^{(n)}$ at integer time is mapped forward by one year by $S^{(n+1)}=f(S^{(n)})$, where

$$\begin{aligned} f(S^{(n)})=e^{-m} S^{(n)} +b. \end{aligned}$$

Since $|f'(S_0(0))|=e^{-m}<1$, the disease-free periodic solution is stable in the disease-free subspace.

For the next generation matrix method, we start with $\boldsymbol{F}$ and $\boldsymbol{V}$ matrices similar to the constant birth model. Because the disease-free solution $\boldsymbol{x}_0=(0,S_0(t))$ is now time-dependent,

$$ \boldsymbol{F} = \begin{bmatrix}\beta S_0(t)\end{bmatrix}, \boldsymbol{V}=\begin{bmatrix}\mu +m\end{bmatrix} .$$

Since these are diagonal ($1\times 1$) matrices, we use Lemma 2.2 of Wang and Zhao (2008) and find $R_0=\frac{\langle \boldsymbol{F}\rangle }{\langle \boldsymbol{V}\rangle }$, where $\langle \cdot \rangle$ indicates the average of a piecewise continuous function over one period. It follows that

7 $$\begin{aligned} R_0&= \frac{\langle \beta S_0(t)\rangle }{\langle \mu +m\rangle }\nonumber \\&=\frac{\int _{0}^{1} \beta S_0(t)\,\textrm{d}t}{\mu +m}. \end{aligned}$$

Then, from Eq. (7),

8 $$\begin{aligned} R_0&= \frac{\beta }{\mu +m}\int _{0}^{1} \frac{be^{-mt}}{1-e^{-m}}\,\textrm{d}t \nonumber \\&= \frac{\beta b}{m(\mu +m)}. \end{aligned}$$

The expressions we obtain for $R_0$ (Eqs. (5) and (8)) are the same for both of the linear predation models, so the timing of the births does not affect the transmissibility of disease within a population experiencing linear predation. To motivate this, consider $\frac{\textrm{d}I}{\textrm{d}t} = (\beta S - \mu -m)I$ when the disease is newly introduced, i.e., *I* is very small, *S* is near the disease-free solution $S_0(t)$, and $\frac{\textrm{d}I}{\textrm{d}t}$ is close to zero so that *I* can be treated as approximately constant. The sign of the prefactor $(\beta S - \mu -m)$ determines whether an outbreak occurs. With linear predation, the only time-dependent part of the prefactor is *S*, which has an average of $\int _{0}^{1}S_0(t)\,\textrm{d}t = b/m$, matching the disease free equilibrium population from the constant birth case.

In fact, even if the birth rate were population-dependent, $R_0$ would still be the same for pulsed and continuous births when predation is linear. This can be found from the results of Roberts and Kao (1998), although the authors did not mention it. Their model (Roberts and Kao 1998) can be written as

$$\begin{aligned} \frac{\textrm{d}S}{\textrm{d}t}&=\mathcal {b}(N,t)-\beta IS-mS \\ \frac{\textrm{d}I}{\textrm{d}t}&=\beta IS-\mu I-mI, \end{aligned}$$

where $N=S+I$ is the total population and $\mathcal {b}(N,t)$ corresponds to either a continuous birth rate of *B*(*N*(*t*))*N*(*t*) at all times *t* or pulses of size *B*(*N*(*n*))*N*(*n*) at integer times *n*. Here we have reduced their model to no vertical disease transmission, bilinear horizontal transmission, and constant per capita natural mortality. Their per capita birth rate *B*(*N*) is a non-increasing function of population. With continuous births,

$$\begin{aligned} R_0=\frac{\beta K}{\mu +m}, \end{aligned}$$

where *K* is the equilibrium disease-free population (at which $B(K)=m$) (Roberts and Kao 1998). With pulsed births,

$$\begin{aligned} R_0=\frac{\beta K_1\frac{B(K_1)}{m}}{\mu +m}, \end{aligned}$$

where $K_1$ is the population just before a birth pulse and $K_1\frac{B(K_1)}{m}$ is the average population of the disease-free periodic solution (Roberts and Kao 1998).

### Hyperbolic Predation

We next calculate $R_0$ when the predation rate is a hyperbolic function of the prey population (Eqs. (3)-(4)), first considering the case of constant birth rate. Using the next generation matrix method (van den Driessche and Watmough 2002) as before, we obtain

9 $$\begin{aligned} R_0 = \frac{\beta b g^2}{(a-b)[\mu g + c(a - b)]}\;. \end{aligned}$$

The effect of each model parameter can be understood. As in the linear predation model, increasing transmission rate $\beta$ will increase $R_0$, and increasing disease-induced mortality $\mu$ will decrease $R_0$. We are assuming that the maximum predation rate *a* is larger than the birth rate *b* to ensure that the population remains finite in the absence of disease, so $a-b>0$. Therefore, increasing the birth rate will again increase $R_0$.

**Fig. 3:**
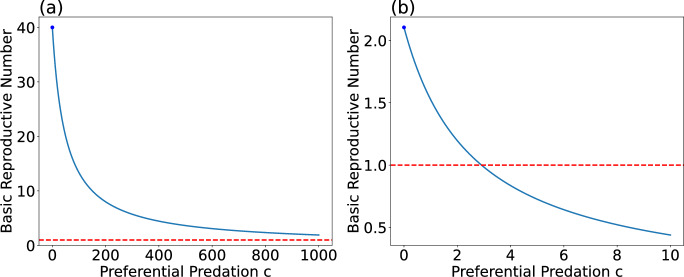
Basic reproductive number $R_0$ (blue) versus preferential predation for hyperbolic predation and constant birth rate when $a=20$ (**a**) and when $a=200$ (**b**). Parameters: $\beta$ = 2, $\mu$ = 5, $g = 100$, $b = 10$. Blue dots indicate $c=0$. Dashed red lines indicates $R_0=1$. (color figure online)

Parameter *c* determines the preference that predators show towards infected individuals. Thus, increasing *c* will decrease $R_0$ by removing more infected individuals from the population. However, the impact depends on whether predation is a significant source of mortality relative to disease mortality. In Fig. 3**a**, the per capita predation rate at small populations is $a/g=0.2$. In comparison, $\mu =5$, causing the system to be more sensitive to disease-induced mortality $\mu$ than to the predation term. Here, $R_0$ cannot be shifted below one with reasonable values of *c*. In Fig. 3**b**, with a much higher maximum predation rate, the disease will die out if $c\gtrapprox 3$, meaning that infected individuals are 3 times as likely to be eaten as susceptible individuals.

The hyperbolic predation model includes two other parameters, *a* and *g*, which we first consider separately. Increasing the maximum predation rate *a* will decrease $R_0$ by leading to fewer infected individuals to spread the disease and fewer susceptible individuals to host it. Increasing the half-saturation parameter *g* will cause the predation function to approach the maximum predation rate more slowly, leading to less predation and therefore a higher $R_0$ value.

We may want to keep $m=a/g$ fixed so that the initial slope of the hyperbolic predation function matches the slope of the linear predation function. Letting $a=mg$, we can rewrite Eq. (9) as

$$\begin{aligned} R_0 = \frac{\beta b}{\left( m-\frac{b}{g}\right) [\mu + c\left( m - \frac{b}{g}\right) ]}\;. \end{aligned}$$

In this case, increasing *g* leads to a smaller $R_0$ value. This can be understood as extending the range of population that can be preyed upon before the predation curve saturates, resulting in more predation.

For the hyperbolic predation model with pulsed births, we use the same technique to find $R_0$ as for the linear predation pulsed birth model. Integrating Eq. (3) for one year, with initial condition $S_0(0)$ immediately after a birth pulse, we find an implicit solution

10 $$\begin{aligned} -\frac{g}{a} \ln {\left[ \frac{S_0(1)}{S_0(0)} \right] } -\frac{1}{a} \left[ S_0(1)-S_0(0) \right] = 1, \end{aligned}$$

where $S_0(1)$ is the susceptible population immediately before the next birth pulse. To have a period 1 solution, also $S_0(1)+b=S_0(0)$. Eq. (10) and the periodicity condition lead to a unique disease-free period 1 solution, which has initial condition

$$\begin{aligned} S_0(0) = \frac{b}{1-e^{(b-a)/g}}. \end{aligned}$$

Although we cannot solve explicitly for $S_0(t)$, Eq. (10) plus a birth pulse implicitly defines a disease-free map $S^{(n+1)}=f(S^{(n)})$ for susceptibles at integer time points. Then

$$\begin{aligned} |f'(S_0(0))| = \frac{S_0(0)^2 +(g-b) S_0(0) -bg }{S_0(0)^2 +(g-b) S_0(0)} <1, \end{aligned}$$

so the disease-free period 1 solution is stable in the disease-free subspace.

We apply the next generation matrix method with

$$ \boldsymbol{F} = \begin{bmatrix}\beta S_0(t)\end{bmatrix}, \boldsymbol{V}=\begin{bmatrix}\mu +\frac{ac}{g+S_0(t)}\end{bmatrix} .$$

Following Lemma 2.2 of Wang and Zhao (2008), we obtain

11 $$\begin{aligned} R_0 = \frac{\beta \int _{0}^{1} S_0(t) \,\textrm{d}t}{\mu +ac\int _{0}^{1} \frac{1}{g+S_0(t)} \,\textrm{d}t}. \end{aligned}$$

Non-linearity of the hyperbolic predation term prevents us from analytically finding a closed-form expression for the disease-free solution $S_0(t)$. In the absence of a closed-form solution, we estimate the value of $R_0$ by solving the integrals in Eq. (11) numerically. We also use a series approximation to find the next order correction to the disease-free solution when populations are small enough that the hyperbolic predation function is close to linear (Appendix A).

### Comparison of the Epidemic Threshold Across Models

**Fig. 4:**
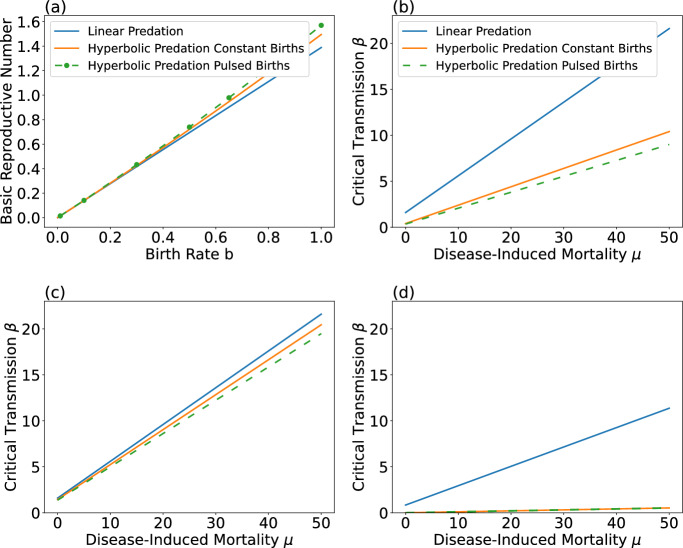
(**a**) $R_0$ versus birth rate *b* for small birth rates. Points on the hyperbolic predation pulsed birth curve indicate where the disease-free solution was found via simulation. Parameters: $a = 20$, $\beta = 50$, $g = 5$, $\mu = 5$. (**b**)-(**d**) Critical transmission $\beta$ versus disease-induced mortality $\mu$ for varying values of *a*, *g*, and *b*. Parameters for (**b**) are $a = 20$, $g = 5$, $b = 10$. Parameters for (**c**) are $a = 200$, $g = 50$, $b = 10$. Parameters for (**d**) are $a = 20$, $g = 5$, $b = 19$. In all panels, $c = 1$, and $m = a/g$ (color figure online).

Fig. 4 compares all four model versions in terms of infection endemicity. The basic reproductive number $R_0$ is shown in Fig. 4**a**. Fig. 4**b**-**d** show the critical transmission $\beta$ value, defined as the transmission rate where the disease-free state becomes unstable and the infection starts to spread. We find the critical $\beta$ values by solving for $\beta$ when $R_0=1$.

With linear predation, $R_0$ does not depend on the timing of births, making linear predation equally effective at controlling disease whether births occur year round or in pulses. Due to the higher predation rate, it is associated with less disease spread (lower $R_0$ and higher critical transmission rate $\beta$) than is hyperbolic predation. The difference becomes more noticeable at higher birth rates *b* (Fig. 4**a**,**d**) and at smaller half-saturation constants *g* (compare Fig. 4**b** to **c**), both of which make hyperbolic predation less effective.

While linear predation is more effective at controlling disease spread overall, nonlinear predation means that the timing of births can affect whether disease spreads. We expect the timing to be less important in two limits: when the population stays small so the hyperbolic predation function is approximately linear, and when the population stays large so the hyperbolic function is approximately constant.

For hyperbolic predation, it is helpful to compare the average population in the disease-free state with pulsed births, $\langle S_0(t) \rangle _\text {pulsed}$, to the disease-free equilibrium with constant births, $S_{0,\text {constant}}$. At small birth rates, the relative difference between them is

12 $$\begin{aligned} \frac{\langle S_0(t) \rangle _\text {pulsed}-S_{0,\text {constant}}}{S_{0,\text {constant}}} = \frac{b}{a} \left[ \frac{a}{2g} \coth {\left( \frac{a}{2g}\right) } -1 \right] \end{aligned}$$

to lowest order in *b* (Appendix A). It can be checked numerically that the quantity $x\coth {x}-1$ is positive and increasing with *x* for $x>0$. This means that pulsed births result in larger average populations than do constant births. Predators become overwhelmed after a birth pulse and are less effective at controlling the population. The relative difference increases in magnitude as the birth rate *b* increases or as the half-saturation constant *g* decreases, i.e., as the hyperbolic predation rate is more nonlinear over the range that the population oscillates when births are pulsed. In Fig. 4**a**, the deviation between pulsed and constant birth $R_0$ increases as the birth rate increases. Although this is not visible by eye for the critical transmission rates in Fig. 4**b**-**c**, numerical calculations showed that the relative difference between pulsed and constant births is larger in **b** which uses a smaller *g* value (keeping fixed the initial slope of the predation curve, $\frac{a}{g}$).

Figure 4**a**-**c** demonstrate that the timing of births has a lower impact on disease spread when small populations cause the hyperbolic predation function to be approximately linear. In Fig. 4**d**, we see another situation in which the disease spread does not depend strongly on the timing of births. Here, the birth rate *b* is almost as large as the maximum predation rate *a*. The resulting large population causes the hyperbolic predation rate to stay in the saturated portion of the curve. Then the predation rate is almost constant despite the pulses of new individuals, and the hyperbolic pulsed and constant birth models are very similar.

## Endemic Infection

In this section, we consider system behavior when infection is endemic. We are especially interested in the pulsed birth case because it produces time-dependent infection levels.

### Dynamical Behavior

**Fig. 5:**
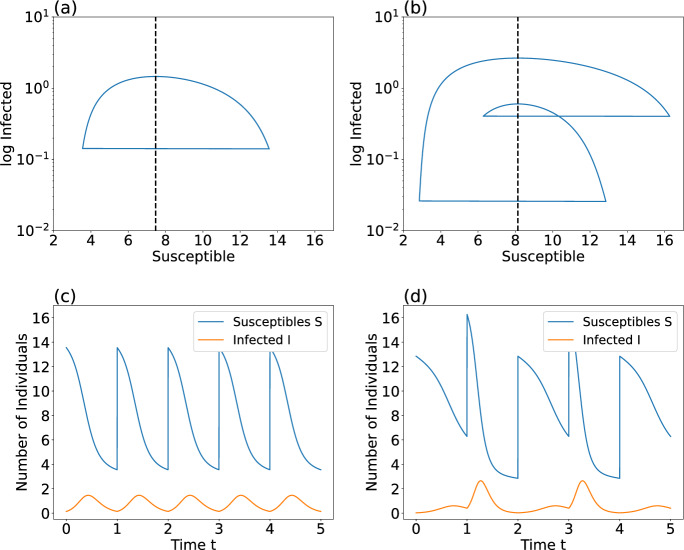
(**a**) and (**b**) show periodic solutions in the susceptible-infected plane for linear predation simulations where $\mu$ = 11 in (**a**) and $\mu$ = 12 in (**b**). The corresponding time series for (**a**) is shown in (**c**) and (**b**) is shown in (**d**). Other parameters: $\beta$ = 1.5, $b = 10$, $m = 0.2$. Dashed lines in (**a**) and (**b**) represent the susceptible population thresholds (color figure online).

Annual birth pulses can result in period 1 solutions, as shown in Fig. 2 and Fig. 5**a**,**c**. The models are not restricted to this behavior, however, and can exhibit longer period behavior depending on parameters and initial conditions. A period 2 solution is shown for the linear predation model in Fig. 5**b**,**d**, and even longer periods, including period 6, have been observed, especially at larger values of the disease mortality rate $\mu$. The dynamics of the system is rich, with multiple stable periodicities coexisting at some parameter values. We observed longer period behavior and coexisting periodicities in both the linear and hyperbolic predation pulsed birth models, indicating that these behaviors are not limited by the type of predation.

Period 1 and period 2 solutions for similar parameter values are compared in Fig. 5. Focusing on the linear predation model, we see that the prefactor $(\beta S - \mu - m)$ from Eq. (2) must be positive for infection to spread. Therefore, there is a susceptible population threshold at $S=\frac{m+\mu }{\beta }$ where susceptible populations greater than this threshold value result in an infection outbreak. In Fig. 5**a**,**b**, a birth pulse brings the susceptible population above this threshold and the infected population subsequently increases. Susceptible populations that are farther above the threshold convert more individuals into infected individuals by the time the susceptible population drops below the threshold, resulting in a larger outbreak. The smallest outbreaks in Fig. 5 occur in 5**b** when a birth pulse brings the susceptible population somewhat past the threshold, causing the infection to spread slightly within the population. However, there is not a large enough outbreak to bring the susceptible population down to the level it was before the birth pulse. The subsequent birth pulse then increases the susceptible population farther beyond the threshold, triggering a large outbreak. On the other hand, in Fig. 5**a** a single birth pulse increases the susceptible population far enough past the threshold for a moderate outbreak, which decreases the susceptible population to the level it was before the birth pulse, resulting in period 1 behavior. We anticipate that parameter values that contribute to low susceptible and infected numbers at the end of an outbreak (as in the lower left of Fig. 5**b**) will promote longer period behavior. Observing more long period behavior at high disease mortality $\mu$ is consistent with this idea.

We next consider the average number of infected individuals over time, focusing specifically on linear predation. We already saw that the basic reproductive number $$ R_0$$ is the same whether births occur constantly or in pulses (Section 3.1), and we will now show that the average infection level is also the same. This result can be found by comparing the endemic equilibrium solution of the constant birth model and an endemic periodic solution of the pulsed birth model.

We start with the constant birth case in which $\mathcal {b}(t)=b$. The endemic equilibrium of Eqs. (1)-(2) requires that

13 $$\begin{aligned} \begin{aligned} b- mS - (\mu +m)I&=0 \\ \beta S-(\mu +m)&=0, \end{aligned} \end{aligned}$$

where the first equation comes from adding Eqs. (1)-(2) and the second from dividing Eq. (2) by *I*. The solution to Eq. (13) is $S_*=\frac{\mu +m}{\beta }$ and $I_*=\frac{b}{\mu +m}-\frac{m}{\beta }$.

Now consider the pulsed birth case where $\mathcal {b}(t)$ is periodic with period *T*. Eqs. (1)-(2) (with $I\ne 0$) imply

14 $$\begin{aligned} \frac{\textrm{d}}{\textrm{d}t}(S+I)= &  \mathcal {b}(t)- mS - (\mu +m)I \end{aligned}$$

15 $$\begin{aligned} \frac{\textrm{d}}{\textrm{d}t}(\ln {I})= &  \frac{1}{I} \frac{\textrm{d}I}{\textrm{d}t} = \beta S-(\mu +m). \end{aligned}$$

Assume the solution (*S*(*t*), *I*(*t*)) is periodic with period *nT*, where $n\in \mathbb {Z}_+$. Then $S(nT)+I(nT)-S(0)-I(0) = 0$ and $\ln {I(nT)}-\ln {I(0)} = 0$. Hence

16 $$\begin{aligned} \begin{aligned} S(nT)+I(nT)&-S(0)-I(0) = \int _{0}^{nT} \frac{\textrm{d}}{\textrm{d}t}(S+I) \,\textrm{d}t \\&= \int _{0}^{nT} \mathcal {b}(t) \,\textrm{d}t - m\int _{0}^{nT} S(t) \,\textrm{d}t -(\mu + m) \int _{0}^{nT} I(t) \,\textrm{d}t \\&= 0 \end{aligned} \end{aligned}$$

and

17 $$\begin{aligned} \begin{aligned} \ln {I(nT)}-\ln {I(0)}&= \int _{0}^{nT} \frac{\textrm{d}}{\textrm{d}t}(\ln {I}) \,\textrm{d}t \\&= \beta \int _{0}^{nT} S(t) \,\textrm{d}t - nT(\mu +m) = 0. \end{aligned} \end{aligned}$$

Dividing Eqs. (16) and (17) by 1/*nT*, and recalling that *b* is the average number of births per year in the pulsed birth case, we see that

18 $$\begin{aligned} \begin{aligned} b- m \bar{S} - (\mu +m)\bar{I}&=0 \\ \beta \bar{S}-(\mu +m)&=0, \end{aligned} \end{aligned}$$

where we are defining $\bar{S}=\frac{1}{nT}\int _{0}^{nT} S(t) \,\textrm{d}t$ and $\bar{I}=\frac{1}{nT}\int _{0}^{nT} I(t) \,\textrm{d}t$, the average numbers of susceptible and infected individuals, respectively, over one period. Because the average values $\bar{S}$ and $\bar{I}$ satisfy the same equations (Eq. (18)) as the equilibrium points $S_*$ and $I_*$ (Eq. (13)), and these equations have a unique solution, both linear predation models have the same average number of infected individuals. Note that this result holds even for longer period solutions.

### Comparison of Infection Level Across Models

**Fig. 6:**
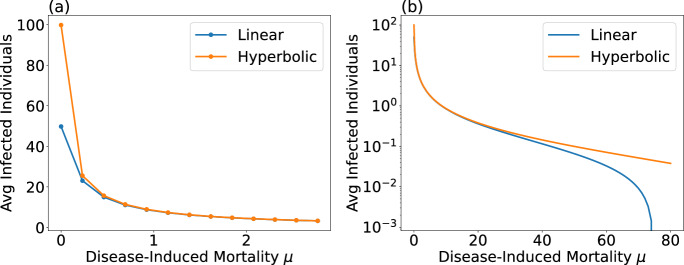
Average number of infected individuals over one period versus disease-induced mortality $\mu$ for linear and hyperbolic predation with pulsed births. (**a**) uses a linear scale on the vertical axis while (**b**) uses a log scale. Parameters: $\beta$ = 1.5, $m=0.2$, *a* = 20, *g* = 100, *b* = 10, $c=1$.

We have already shown that hyperbolic predation leads to a larger $R_0$ than linear predation does. Focusing on the pulsed birth case, we will now examine how the predation function affects endemic infection levels. We find the average infection level over one period by numerically integrating with the trapezoid rule using the SciPy trapz function. Again, we expect the biggest difference between models when the population is large enough for the hyperbolic function to approach saturation. In Fig. 6, both models have similar infection levels when disease-induced mortality $\mu$ is high, keeping the population low. However, hyperbolic predation results in more infection when disease-induced mortality $\mu$ is low (Fig. 6**a**). When $\mu =0$, the average total population in the hyperbolic predation model is approximately equal to the half-saturation constant *g*; therefore, the predation rate is approaching saturation and the predators cannot control the disease as well. With very rapid disease mortality, infection levels are of course low, but the hyperbolic predation model has an endemic state at higher $\mu$ than the linear predation model because of its larger $R_0$ (Fig. 6**b**).

Next, we consider how the birth process interacts with hyperbolic predation. When births occur constantly, the population reaches an equilibrium and the predation rate is constant. However, when births occur in pulses, the population oscillates periodically, accessing different parts of the nonlinear predation curve.

**Fig. 7:**
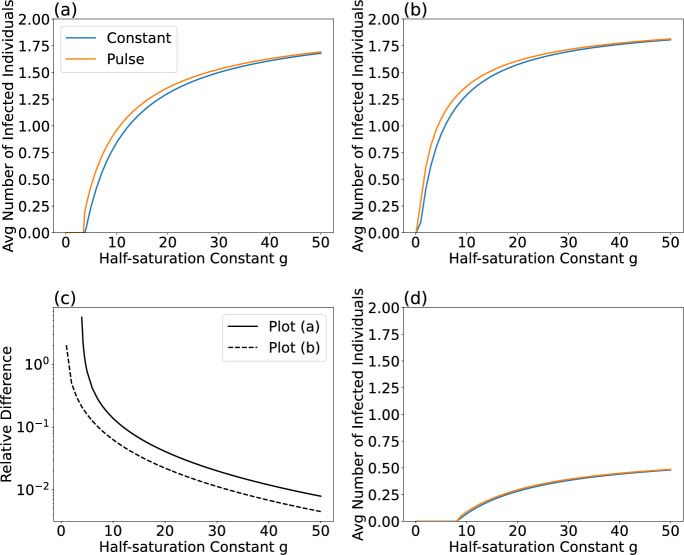
(**a**) ,(**b**), (**d**) Show the average number of infected individuals over one period versus the half-saturation constant *g* for the hyperbolic predation models. In all panels, $b=10$, $c=1$, $\beta = 2$. Parameters for (**a**) are $a = 20$ and $\mu = 5$. There is a lower maximum predation rate in (**b**). Parameters for (**b**) are $a=12$ and $\mu = 5$. There is a higher disease-induced mortality value in (**d**). Parameters for (**d**) are $a = 20$ and $\mu = 15$. (**c**) shows the relative difference between infection levels with constant and pulsed births for (**a**) and (**b**) (color figure online).

There is a greater difference between infection levels of the pulsed and constant birth models when there is more death due to predation. Because of a higher maximum predation rate, individuals are dying more from predation in 7**a** than in 7**b**. So, the nonlinearity of the predation function has a greater effect on average infection in 7**a**, resulting in a somewhat greater difference between the constant and pulsed birth models. When the maximum predation is decreased, disease mortality is more important, the predation function has less of an effect on the system, and the two curves are somewhat closer together (Fig. 7**b**). Fig. 7**c** shows the relative difference between the pulsed and constant birth models for both **a** and **b**, conveying that the relative difference is lower when *a* is lower.

Also seen in Fig. 7**c** is that the relative difference between the pulsed and constant birth models decreases as the half-saturation constant *g* increases. Larger *g* both reduces predation and makes the linear regime of the hyperbolic predation function span a wider range of populations. In absolute terms, the effect of oscillating population on infection levels is most noticeable at moderate *g*–above the epidemic threshold but not too large (Fig. 7**a** and **b**).

Finally, Fig. 7**d** shows a higher disease-induced mortality rate $\mu$. Even for low *g* values when the population accesses the nonlinear regime of the hyperbolic function, the impact of disease mortality outweighs the impact of predation, so the constant and pulsed birth models are very similar.

## Conclusion

In this paper, we defined an SI model to study how seasonal births and nonlinear predation (or other negative density-dependent mortality) interact with infection spread. Specifically, we considered constant versus pulsed births and linear versus hyperbolic predation functions. For the hyperbolic case we included the possibility of preferential predation, where infected individuals are disproportionately more or less likely to die from predation. We obtained analytic expressions for the basic reproductive number $R_0$ for all cases except pulsed births with hyperbolic predation and compared disease dynamics across models through simulation.

When predation mortality was linear in prey density, the timing of births (pulsed versus constant) did not affect the basic reproductive number $R_0$ nor the average endemic infection level. Even with a density-dependent birth function, linear mortality leads to $R_0$ that is independent of birth timing (Roberts and Kao 1998). This result, while perhaps surprising, is encouraging for those who prefer simple models. In such cases, the timing of births need not be represented carefully if only the average infection level is needed (e.g., Al-Arydah et al. (2016)). However, the timing of births can matter when predation mortality is nonlinear. Hyperbolic predation sometimes resulted in more infection spread when births occurred in pulses rather than constantly. This was mainly noticeable at intermediate populations where the curvature of the hyperbolic function was the greatest. Even so, the differences we saw due to the birth process were not extremely large, perhaps again suggesting that birth timing need not always be modeled in detail (e.g., Fitzgerald and Keener (2025)). Likewise, Roberts and Kao (1998) previously observed that with more complicated density-dependent birth and death functions, $R_0$ for tuberculosis in possums was still very close for continuous and pulsed births.

Disease spread occurred more easily when predation saturated at high prey densities. Lower predation mortality kept susceptible and infected populations higher and allowed more disease transmission. As expected, this resulted in higher $R_0$ and higher endemic infection levels for hyperbolic predation than for linear. Preferential predation on infected individuals could lower $R_0$, but the magnitude of the effect depended on whether predation was a significant source of mortality relative to disease-induced mortality.

A major question in predator-prey studies is how predation affects disease levels, with the common “Healthy Herds Hypothesis” being that predation will reduce infection prevalence (Packer et al. 2003; Richards et al. 2022). Our model behaved consistently with that idea, with both $R_0$ and the average number of infected individuals decreasing as predation increased. Disease spread occurred more easily when predation saturated at high prey densities, resulting in higher $R_0$ and higher endemic infection levels for hyperbolic predation than for linear. However, in more complex situations, increases in disease under predation may arise from either direct biological mechanisms or emergent system dynamics. The possibility of non-host predators dispersing the disease (“predator-spreader hypothesis”) (Cáceres et al. 2009) is a major factor in whether predators will increase infection prevalence (Richards et al. 2022). Thus, it is important to understand the predator feeding biology. Alternatively, increased infection prevalence can be an emergent property of models with predation. Roy and Holt (2008) found that even in a simple model with the predators as a fixed parameter, infection abundance and prevalence can depend non-monotonically on predation due to interactions between the predator and recovered prey. Further, infection prevalence can vary non-monotonically in cases where $R_0$ strictly decreases with predation (Roy and Holt 2008). When predator population is modeled explicitly, a specialist predator may require an alternative food source to maintain its population and effectively control disease in prey (Sahoo and Poria 2016), a phenomenon that could not occur in our model with the predators as a fixed parameter.

Another area of longtime interest in predator-prey systems is the possibility of oscillations, which can already occur in the absence of disease and seasonality (Wangersky 1978; Diz-Pita and Otero-Espinar 2021). Such oscillations are not expected in our disease-free model since our constant birth rate is equivalent to steady immigration; even a small immigration rate of prey has been found to prevent oscillations (Sugie and Saito 2012; Tahara et al. 2018). In contrast, population-dependent birth rates are much more common in eco-epidemiological models (Gómez-Hernández et al. 2024). Another source of cyclic behavior is that infection and hyperbolic predation can combine to generate oscillations (Roy and Holt 2008). This effect can occur even with predators as a fixed parameter and is mediated by the degree to which the predator prefers infected prey (Hall et al. 2005). An interesting area for future study is how the oscillations present in nonseasonal predator-prey-disease models can interact with seasonal births. Compared to the model in this paper, density-dependent births seem likely to promote longer period solutions because the susceptible population will take longer to build up again after high mortality due to an outbreak. However, interactions between different sources of oscillations will probably depend on how disease transmission is modeled. Frequency-dependent transmission (transmission rate proportional to the fraction of infected individuals rather than to the number of infected individuals as we have assumed here) can instead have a stabilizing effect and reduce oscillations (Hilker and Schmitz 2008; Gómez-Hernández et al. 2024).

We considered linear and hyperbolic functional responses (Holling type I and type II) in this paper. Another future direction is to model sigmoidal predation (Holling type III, Holling (1965)). A sigmoidal functional response can occur due to prey switching. Low prey availability leads predators to use a more abundant food source. As the prey population increases, predators switch to the species of interest and the predation rate per prey increases, but the predation rate saturates at high prey population. In contrast to our hyperbolic predation function, which is close to linear at low prey population, sigmoidal predation is nonlinear even at low prey population. Therefore, a sigmoidal functional response may cause the disease levels to be more sensitive to population changes and the timing of new births.

## Data Availability

The code and simulation data associated with this paper are available on GitHub at https://github.com/aintrone/Effects-of-Seasonal-Births-and-Predation-on-Disease-Spread.git

## Appendix A Series Expansion of Disease-Free Solution for Hyperbolic Predation

To obtain the disease-free periodic solution with hyperbolic predation and birth pulses of *b* new individuals per year, we need to solve

A1 $$\begin{aligned} \frac{\textrm{d}y}{\textrm{d}t} = -\frac{ay}{g+y} \end{aligned}$$

for population *y*(*t*) satisfying periodicity condition $y(1)+b=y(0)$. Here we denote population by *y*(*t*) instead of $S_0(t)$ for brevity. Assuming the birth rate $b\ll 1$ is a small parameter, we seek a series solution

A2 $$\begin{aligned} y(t)=y_0(t)+by_1(t)+b^2y_2(t)+\ldots \end{aligned}$$

satisfying condition

A3 $$\begin{aligned} y_0(1)+b[y_1(1)+1]+b^2y_2(1)+\ldots = y_0(0)+by_1(0)+b^2y_2(0)+\ldots . \end{aligned}$$

To second order in *b*, the disease-free solution for $0\le t<1$ is then

A4 $$\begin{aligned} y(t) \approx b \frac{e^{-at/g}}{1-e^{-a/g}} + b^2 \frac{e^{-at/g}}{g(1-e^{-a/g})^2}(1+e^{-a/g}-e^{-at/g}). \end{aligned}$$

Of note, if we instead assume that the half-saturation constant *g* is large, so that $\epsilon = \frac{1}{g}\ll 1$ is a small parameter, and further assume that $\frac{a}{g}$ is held fixed (so $a=mg$ where *m* is a constant), the series solution to order $\epsilon$ is identical to that obtained in Eq. (A4). Unsurprisingly, the lowest order term in Eq. (A4) matches the solution with linear predation obtained in Eq. (6) if $m=a/g$, i.e., when the initial slope of the hyperbolic predation function matches the slope of the linear predation function. With hyperbolic predation, the population can be kept low enough that the predation rate does not differ much from linear if either the birth rate *b* is small or the half-saturation constant *g* is large. Either method of keeping the hyperbolic predation rate almost linear over the whole population range will result in the same next order correction to the linear predation solution.

The average number of susceptible individuals in the disease-free state can be computed using Eq. (A4) as

A5 $$\begin{aligned} \langle S_0(t) \rangle _\text {pulsed} = \int _0^1 y(t) dt \approx \frac{bg}{a} +\frac{b^2}{2a}\frac{1+e^{-a/g}}{1-e^{-a/g}} \end{aligned}$$

to $\mathcal {O}(b^2)$. On the other hand, the disease-free equilibrium with constant births and hyperbolic predation is

A6 $$\begin{aligned} S_{0,\text {constant}} = \frac{bg}{a-b} \approx \frac{bg}{a}+\frac{b^2g}{a^2}. \end{aligned}$$

The effect of pulsed births on average population in the context of hyperbolic predation can be seen from the relative difference between Eqs. (A5) and (A6):

A7 $$\begin{aligned} \frac{\langle S_0(t) \rangle _\text {pulsed}-S_{0,\text {constant}}}{S_{0,\text {constant}}} = \frac{b}{a} \left[ \frac{a}{2g} \coth {\left( \frac{a}{2g}\right) } -1 \right] . \end{aligned}$$
